# Occurrence of pathogenic *Vibrio parahaemolyticus* in crustacean shellfishes in coastal parts of Eastern India

**DOI:** 10.14202/vetworld.2016.330-336

**Published:** 2016-03-28

**Authors:** S. Parthasarathy, Suresh Chandra Das, Ashok Kumar

**Affiliations:** 1 Division of Veterinary Public Health, Indian Veterinary Research Institute, Izatnagar, Bareilly - 243 122, Uttar Pradesh, India; 2 Veterinary Public Health Laboratory, Indian Veterinary Research Institute, Eastern Regional Station, Kolkata - 700 037, West Bengal, India; 3 Assistant Director General (Animal Health), Indian Council of Agricultural Research, Krishi Bhawan, New Delhi, India

**Keywords:** crustaceans, pandemic, pathogenic, *toxR*-gene, *Vibrio parahaemolyticus*

## Abstract

**Aim:**

The objective of the study was to isolate and characterize pathogenic *Vibrio parahaemolyticus* from crustacean shellfishes (crab and shrimp) commonly retailed in coastal parts of eastern India.

**Materials and Methods:**

Samples were processed by bacteriological isolation followed by biochemical characterization in Kaper’s medium. Presumptively identified isolates were confirmed by species-specific Vp-*toxR* polymerase chain reaction (PCR) assay. Virulence and pandemic property of the confirmed *V. parahaemolyticus* isolates were determined by specific PCR assays.

**Results:**

On screening of 167 samples comprising crabs (n=82) and shrimps (n=85) by the standard bacteriological cultural method, *V. parahaemolyticus* was presumptively identified in 86.6% (71/82) and 82.3% (70/85) of respective samples. Of these, 46 (56%) and 66 (77.6%) isolates from crab and shrimp, respectively, were confirmed as *V. parahaemolyticus* by biochemical characterization (Kaper’s reaction) followed by specific Vp-*toxR* PCR assay. About 10 isolates each from crab and shrimp was found to carry the virulence gene (*tdh*). It denotes that 12.2% of crab and 11.7% of shrimp in the study area are harboring the pathogenic *V. parahaemolyticus*. Such *tdh*^+^ isolates (n=20) were subjected for screening of pandemic genotype by pandemic group specific (PGS) - PCR (PGS-PCR) and GS-PCR (*toxRS* gene) where 11 (6.5%) isolates revealed the pandemic determining amplicon (235 bp) in PGS-PCR and belonged to crab (7.3%) and shrimp (6%) samples; however, 2 (2.4%) isolates were positive in GS-PCR and belonged to crab samples only. These two GS-PCR^+^ isolates from crab were also positive in PGS-PCR.

**Conclusion:**

The findings of the study conclusively indicated that a considerable percentage of crab and shrimp in these areas were harboring pathogenic and pandemic *V. parahaemolyticus* posing a public health risk in consumption of improperly processed such shellfishes. Cross contamination of other marine and fresh water market fishes by such shellfishes in these areas may provide scope for spreading this pathogen in community food chain.

## Introduction

Globalization of the food supply and increased international travel has enhanced the risk of occurrence of different diseases in many parts of the world. Moreover, changes in nutritional habits brought about an increase in consumption of undercooked or raw foods, especially sea foods such as marine fish and shellfish implicated with *Vibrio parahaemolyticus* exposing consumers to different diseases more specifically gastroenteritis and diarrhea [1]. Occupying a variety of niches, *V. parahaemolyticus* is a common bacterium in marine and estuarine environments [2]. It can exist planktonically or attached to submerged inert and animate surfaces, including suspended particulate matter, zooplankton, fish and shellfish [3]. It belongs to genus *Vibrio* and commonly isolated from various seafoods including oyster, mussel, scallop, octopus, shrimp, clam, crab, mackerel, sardines, codfish, etc., worldwide [4]. This organism is recognized globally as one of the leading causes of food poisoning (toxi-infection), diarrhea and gastroenteritis in human resulting from the consumption of raw or insufficiently cooked seafood [1,5]. Thermostable direct hemolysin (TDH) and TDH-related hemolysin (TRH), encoded by the *tdh* and *trh* genes, respectively, have been recognized as the major virulence factors of this organism [6,7]. TDH causes β-hemolysis of human erythrocytes in Wagatsuma agar medium, popularly known as the Kanagawa phenomenon [8].

West Bengal and Odisha, the two eastern coastal states of India are important hub for the harvesting of marine fish and shellfishes. The average brackish water fish production was around 30,000 MT which includes 5000 MT of shrimp and 350 MT of crab harvested from the Chilika Lake, the important saltwater fishing harbor in Odisha (Directorate of Fisheries, Government of Odisha; website link: http://www.odishafisheries.com). The export of marine products to foreign countries, such as Japan, Thailand, and Indonesia, is about 30,900 MT which costs around 1800 crores annually (Directorate of Fisheries, Government of Odisha). West Bengal is the only state in India, where fishes have been cultivated in all types of water bodies’, i.e., sweet water, brackish water, sewage water, and marine water, etc. The total productions of inland fish and marine fish in WB are 15.30 Lac ton and 2 Lac ton, respectively. These are mainly consumed in the state and rest spare for Delhi, Uttar Pradesh, Madhya Pradesh, Bihar, and other adjoining states. Export of marine fish earned handsome revenue of Rs.700 crore in the year 2009-2010. West Bengal occupies the 4^th^ position in the country regarding export of seafood products. Fishes are exported primarily to Japan, Vietnam, and China. Out of the total exports, 90% are shrimps and the rest includes ornamental fish, crab, fresh water prawns (Food Processing Industries Survey, West Bengal, 2012-13; website link: http://www.wbfpihgov.in).

Ingestion of raw or improperly cooked seafoods, mainly crustacean and molluscan shellfishes have been identified the main sources of *V. parahaemolyticus* infections, and this has emerged as a growing concern in the production and trade of seafoods [1,2]. Kolkata, an inland metropolis is an endemic area for diarrheal diseases and *V. parahaemolyticus* was detected from 3.5% to 23.9% of acute human diarrheal cases [9]. Since 1996, the incidence of *V. parahaemolyticus* associated infections has increased with an emergence of highly virulent pandemic O3: K6 clone [10]. On the other hand, magnitude of occurrence of this organism has not been properly addressed in coastal Odisha, i.e. in and around Bhubaneswar, India. These marine shellfishes, such as crab and shrimps, provide an affordable protein dishes in this geographical region that replaces more than 30% of the local fish consumption and are suspected as a potential source of diarrheal diseases. Further, in Indian context, the past studies, so far, reported the incidence of this organism mostly from clinical diarrhea [11-13] and a few studies from marine fishes [14-16] but not from the different shellfishes retailed in suburban and proper Kolkata and Bhubaneswar areas. Keeping this in view, the present investigation was undertaken to determine the occurrence of pathogenic *V. parahaemolyticus* in shellfishes namely crabs and shrimp in these areas as well as to identify their pandemic population.

## Materials and Methods

### Ethical approval

In this study, samples from saline water shellfishes viz, crab and shrimp were collected from retail market and examined for presence of the organism by cultural isolation and thereafter, characterized for their virulence traits in the laboratory adopting the recommended assays where no animal experiment was involved.

### Design of study

The study was designed for isolation and identification of *V. parahaemolyticus* from saline water origin shellfishes such as crab and shrimp by bacteriological isolation and biochemical characterization followed by confirmation in species-specific Vp-*toxR* polymerase chain reaction (PCR) assay. The confirmed isolates were further characterized for their pathogenic and pandemic property by molecular characterization employing specific PCR methods.

### Study area

**Figure-1:**
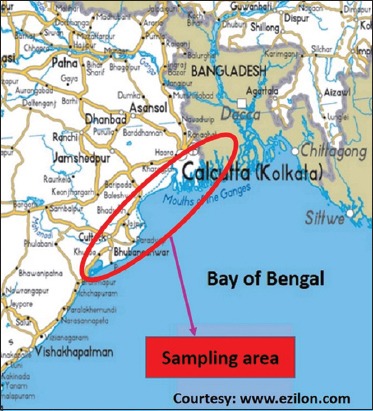
Location of sampling areas.

A total of 167 samples of crab (n=82) and shrimp (n=85) collected aseptically in sterile sample container from different retail fish markets in and around the city of Kolkata and Bhubaneswar, India (Figure-1) and transported in ice packs to the laboratory for further processing.

### Sample processing

Intestinal mass of crab and head portions of shrimp were considered for processing. About 10-20 g of sample was aseptically grinded using pestle and mortar. The masses were inoculated in 50 ml of alkaline peptone water containing 3% NaCl at pH 8.5 (pre-enrichment) and incubated aerobically for 24 h at 37°C. During processing, all necessary precautions were taken to avoid cross-contamination of the samples.

### Cultural isolation

Isolation and identification of *V. parahaemolyticus* were carried out by adopting standard bacteriological methods [17] with minor modifications. Briefly, a loop full of overnight broth was streaked on thiosulfate citrate bile salts sucrose agar and the plates were incubated at 37°C for 24 h. Presumptive identification of *V. parahaemolyticus* was carried out based on typical colony characteristics, i.e., round, 2-3 mm in diameter with green or blue center. Five typical colonies from each plate (each sample) were selected for biochemical characterization in the Kaper’s multi test medium where characteristic colonies of the *V. parahaemolyticus* revealed acidic (yellow) butt and alkali (purple) slant (K/A) reaction. Such characteristic colonies (n=112; Table-1) were subjected to screen for species-specific *toxR* gene by Vp-*toxR* PCR assay.

**Table-1:** Occurrence of *V. parahaemolyticus* in shellfishes.

Samples screened (n)	Cultural isolation in TCBS (%)	Biochemical characterization (Kaper’s Reaction) (%)	PCR assay			

			Species confirmation (Vp-*toxR*) (%)	Virulence characterization (%)				

				*tdh*	GS-PCR	PGS-PCR
Crab (82)	71 (86.6)	46 (56)	46 (56)	10 (12.2)	2 (2.4)	6 (7.3)
Shrimp (85)	70 (82.3)	66 (77.6)	66 (77.6)	10 (11.7)	0	5 (6)
Total (167)	141 (84.4)	112 (67)	112 (67)	20 (11.9)	2 (1.2)	11 (6.5)

TCBS=Thiosulfate citrate bile salts sucrose, PGS=Pandemic group specific, PCR=Polymerase chain reaction, *V. parahaemolyticus=Vibrio parahaemolyticus*

### Confirmation of isolate by species-specific Vp-*toxR* PCR assay

**Figure-2:**
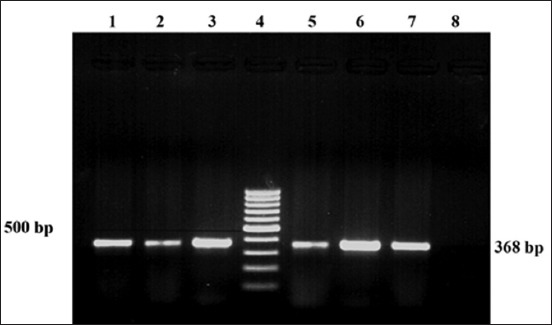
Polymerase chain reaction amplification of *toxR* gene of *Vibrio parahaemolyticus*, Lane 1, 2, 3, 5, 6: Sample DNA (C2, C25, C57, S8, S38) with positive amplicon (368 bp), Lane 4: DNA ladder of molecular weight 100 bp, Lane 7: Positive control (*V. parahaemolyticus* Vp-Kx-V_138_ strain), Lane 8: Negative control, C - Crab, S – Shrimp.

The Vp*-toxR* PCR assay was standardized for detection of species-specific *toxR* gene of *V. parahaemolyticus* adopting the described method [18] with some modification using the bacterial lysate as template DNA prepared from the lawn culture on LB agar plates by snap chill method. Briefly, a loopful of fresh bacterial culture was mixed with 100 µl Mlli Q water in a microcentrifuge tube and centrifuged at 10,000 rpm for 5 min. The tube was kept in a boiling water bath for 10 min. After heat treatment, the cell lysate was immediately kept in ice cubes. After 10 min, it was centrifuged at 6000 rpm for 5 min and supernatant was used as template DNA. The primers as mentioned in Table-2 were used in this assay. Amplification reaction was performed in 25 µl reaction volume containing 2.5 µl 10× PCR amplification buffer (500 mM KCl, 100 mM Tris-HCl, pH-8.3; 15 mM MgCl_2_), 0.5 µl dNTP mix (10 mM each), 1 µl (10 pmol/µl) each of forward and reverse primers, 0.2 µl (1 unit) Taq DNA polymerase, 5.3 µl of 1:10 diluted bacterial lysate and sterile deionized water to make volume up to 25 µl. Cycling conditions include initial denaturation at 95°C for 5 min followed by 20 cycles of denaturation (94°C for 1 min), annealing (63°C for 1.30 min) and extension (72°C for 1.30 min) and final extension was carried out at 72°C for 7 min. The amplified product (368 bp) was electrophoresed on 1.5% agarose gel, visualized under ultraviolet (UV) light after staining with ethidium bromide (0.5 µg/ml), and the result was recorded comparing with reference strain Vp-Kx-V138 (Figure-2).

**Table-2:** Oligonucleotide primers used in different PCR assays.

PCR assay	Target gene	Primer sequences (5`-3`)	Amplicon	References
*Vp-toxR* PCR	*tox*R	F: GTC TTC TGA CGC AAT CGT TG R: ATA CGA GTG GTT GCT GTC ATG	368 bp	[18]
*tdh* PCR	*tdh*	F: CCA AAT ACA TTT TAC TTG G R: GGT ACT AAA TGG CTG ACA TC	199 bp	[19]
GS-PCR	*toxRS*/new sequence	F: TAA TGA GGT AGA AAC A R: ACG TAA CGG GCC TAC A	651 bp	[20]
PGS-PCR	PGS sequence	F: TTC GTT TCG CGC CAC AAC T R: TGC GGT GAT TAT TCG CGT CT	235 bp	[21]

PGS=Pandemic group specific, PCR=Polymerase chain reaction

### Detection of virulence gene (*tdh*)

**Figure-3:**
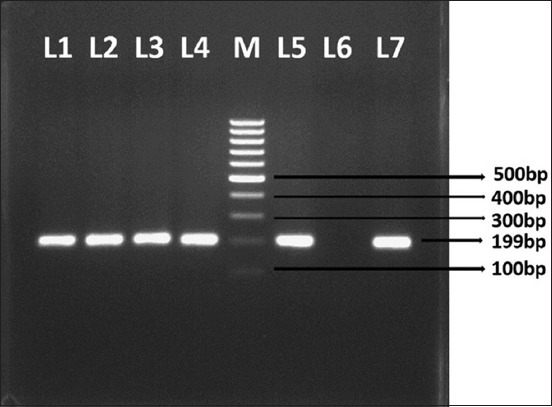
Polymerase chain reaction amplification of *tdh* gene of *Vibrio parahaemolyticus*, Lane 1, 2, 3, 4, 6: Sample DNA (C2, C25, C57, S8, S38) with positive amplicon (199 bp), Lane 5: DNA ladder of molecular weight 100 bp, Lane 7: Negative control, Lane 8: Positive control (*V. parahaemolyticus* Vp-Kx-V_138_ Strain), C - Crab, S – Shrimp.

Virulence of the confirmed isolates was determined by defining the presence of cardinal virulence gene, i.e., *tdh* gene for hemolysin production. The *tdh* PCR assay was accomplished following the method [19] using the primers as mentioned in Table-2. The assay involved 2.5 µl of 10× PCR amplification buffer (500 mM KCl, 100 mM Tris-HCl, pH-8.3; 15 mM MgCl_2_), 0.5 µl of dNTP mix (10 mM each), 1 µl (10 pmol/µl) each of forward and reverse primers, 0.2 µl (1 unit) of Taq DNA polymerase, 5.3 µl of bacterial lysate (1:10 dilution) and sterile deionized water to make final reaction volume up to 25 µl. The reaction mixture was cycled at 94°C for 5 min for initial denaturation, then 30 cycles of denaturation at 94°C for 1.30 min, annealing at 50°C for 1.30 min and elongation at 72°C for 1.30 min followed by final extension for 7 min at 72°C. At the end of reaction, the amplified product (199 bp) was electrophoresed on 1.5% agarose gel, visualized under UV light after staining with ethidium bromide (0.5 µg/ml), and the result was recorded comparing with reference strain Vp-Kx-V_138_ (Figure-3).

### Detection of pandemic marker (*toxRS* new/GS-PCR and pandemic group specific-PCR [PGS-PCR])

All the 20 *tdh*^+^ isolates obtained from crab and shrimp were further subjected to GS-PCR and PGS-PCR assay to determine the presence of pandemic gene by employing the published methods [20,21] using primers as mentioned in Table-2. The reaction mixture was optimized to contain 2.5 µl of 10× PCR buffer (500 mM KCl, 100 mM Tris-HCl, pH-8.3; 15 mM MgCl_2_), 0.5 ml of dNTP mix (10 mM each), 1.0 µl (10 pmol/µl) each of forward and reverse primers, 0.2 µl (1 unit) of Taq DNA polymerase, 5.3 µl of bacterial lysate (1:50 dilution for GS-PCR and 1:10 for PGS-PCR) prepared by boiling and snap chilling method and sterile deionized water to make final volume 25 µl. The reaction was performed in a thermal cycler with preheated lid (lid temp 105°C). The cycling condition for GS-PCR was an initial denaturation at 96°C for 5 min, followed by 25 cycles each of denaturation at 96°C for 1 min, annealing at 45°C for 2 min, elongation at 72°C for 3 min and final extension step at 72°C for 7 min. Similarly, for PGS-PCR assay, the initial denaturation was at 94°C for 5 min followed by 25 cycles each of denaturation at 94°C for 1 min, annealing at 59°C for 1 min, elongation at 72°C for 1 min and final extension step at 72°C for 7 min. On completion of the reaction, the amplified products (651 bp and 235 bp) were held briefly at 4°C, and then, analyzed by agarose (1.2%) gel electrophoresis stained with ethidium bromide (0.5 μg/ml) and visualized under UV transilluminator. DNA ladder of 100 bp (Thermo Fischer Scientific, USA) was used as molecular weight marker. The lysate DNA of reference culture Vp-Kx-V_138_ and *E. coli* K12 were used as positive and negative control, respectively.

## Results

A total of 167 samples from crab (n=82) and shrimp (n=85) were examined for the presence of *V. parahaemolyticus* by cultural isolation and biochemical characterization followed by confirmation of the species by species-specific Vp-*toxR* PCR assay. Of the total 167 samples screened by standard bacteriological cultural method, *V. parahaemolyticus* was presumptively identified in 86.6% (71/82) crab and 82.3% (70/85) shrimp samples, respectively (Table-1). On biochemical characterization in Kaper’s multi test medium with such presumptively identified 71 and 70 isolates from crab and shrimp, 46 (56%) and 66 (77.6%) were produced positive K/A reaction, respectively. All the Kaper’s positive isolates were confirmed as *V. parahaemolyticus* in species-specific Vp-*toxR* PCR assay (Table-1). The identified isolates were characterized by *tdh* PCR assay for the presence of cardinal virulence gene, i.e., *tdh* gene responsible for hemolysin production. Of the total 112 confirmed isolates, 10 isolates each from crab (12.2%) and shrimp (11.7%) was found to carry the virulence gene (*tdh*) (Table-1). Subsequently, such *tdh*^+^ isolates (n=20) were subjected for screening of pandemic genotype by PGS-PCR (235 bp amplicon of the AP-PCR fragment for 930 bp) and GS-PCR (*toxRS*/new sequence) where 11 (6.5%) isolates revealed the pandemic determining amplicon (235 bp) in PGS-PCR and belonged to crab (7.3%) and shrimp (6%) samples; however, 2 (2.4%) isolates were positive in GS-PCR and belonged to crab samples only. These two (2) GS-PCR^+^ isolates from crab were also positive in PGS-PCR.

## Discussion

With the increase in human population and growing demand of more food, the aquatic foods including seafoods has been considered as an alternative source of dietetic protein to meet up the growing need of the community. This situation considered as an important cause for a large number of foodborne diseases including of *V. parahaemolyticus* in food chain [22] that causes gastroenteritis (toxi-infection) associated with the ingestion of contaminated raw or improperly cooked saline water origin fish and shellfishes [1,2]. In spite of the large infective dose (10^7^ to 10^8^), the short generation time (8-9 min) enables the organisms to multiply rapidly at ambient temperatures in foods and facilitate to cause disease. Shellfishes including crab and small shrimps serve a staple dietary protein for non-veg fish eaters in Eastern India coastal areas including suburban and proper Kolkata and Bhubaneswar. In 1996, an abrupt increase in diarrheal cases and isolation of *V. parahaemolyticus* was reported in Infectious Disease Hospital, Kolkata with the emergence of highly virulent pandemic strain O3:K6 [10]. Moreover, the first outbreak of *V. parahaemolyticus* mediated diarrhea was reported in Vellore, Tamil Nadu [10]. Further, in India, the incidence of *V. parahaemolyticus* is reported to have doubled during 1996-2000 [11] fetch its clinical and public health importance. Primarily this organism was considered for study because, since 1996, it has been frequently associated in human diarrheal cases in eastern coastal areas and has gained a new global dimension in its pathogenicity by virtue of its emerging the virulence and pandemic characters. Moreover, this is capable of infecting wide host range of marine animals including marine shellfishes which still remains to be the main source of food borne infection in these areas. The perusal of literature suggested that past studies on occurrence of this pathogen were mainly centered on the clinical cases and very few studies with saline water fishes. However, the occurrence of this pathogen in shellfishes available in coastal areas particularly in Odisha has not been addressed where seafood especially crabs and shrimps are included in the daily dishes by a considerable size of population.

Among all the 167 shellfish samples, 141 (84.4%) yielded characteristic *V. parahaemolyticus* in cultural isolation; however, isolates from 112 (67%) such samples revealed positivity in biochemical characteristics and the species-specific *toxR* gene amplicon (368 bp) in Vp-*toxR* PCR assay thereby confirmed the occurrence of *V. parahaemolyticus*. Further, this organism was recorded in little higher frequency (77.6%) in shrimp than crab (56%) (Table-1). The result recorded the sizeable difference in identification of *V. parahaemolyticus* from samples by traditional cultural isolation and the PCR assay. Incidence (67%) of this pathogen in common shellfishes in these areas revealed its potentials as a food borne problem. The observation for the isolation in this study is in accordance with the earlier published works [15] where marine fishes were sampled. The present findings deferred with the observation of Deepanjali *et al*. [23] who reported *V. parahaemolyticus* in 93.87% of oysters. This difference in occurrence may be attributed to variation in factors for geographical areas and type of sample studied. The study findings were also in concordance with earlier studies that reported the presence of this organism in about 50-70% of seafood [24].

PCR was used to detect *tdh* gene using DNA primers that are specific for encoding TDH to determine the pathogenic population among the confirmed *V. parahaemolyticus*. Out of 167 samples, 20 (11.9%) were identified to be virulent by producing specific amplicon for *tdh* gene recognized for production of β-hemolysis evident on Wagatsuma agar referred as Kanagawa phenomena [25]. Moreover, there was no significant difference in occurrence of *tdh*^+^ isolates in the two sample sources, i.e. crab (12.2%) and shrimp (11.7%). In this study, population of *tdh^+^* *V. parahaemolyticus* was comparatively high than the works of Deepanjali *et al.*, [23] where *tdh*^+^ was recorded in 6.1% isolates in South West coast of India and also to the observation of Sakazaki *et al.*, [8] who reported that 1-2% of environmental samples contain virulent (*tdh*^+^) isolates. The study findings were also in agreement with previous studies [26,27] where *tdh*-positive *V. parahaemolyticus* was recovered in 10%, 11% and 15% of shellfishes such as oyster and shrimps. The high frequency of occurrence of pathogenic (*tdh^+^*) *V. parahaemolyticus* in shellfishes (crab and shrimp) in these areas indicates the potentials of common market shellfishes for causing food-borne gastroenteritis linked to food chain. The present study also highlighted the real magnitude of public health risk in terms of gastroenteritis attributed to routine intake of such improperly processed shellfishes that carry pathogenic *V. parahaemolyticus* in their considerable population (11.9%).

In reality, in Indian context, gastroenteritis to the consumers are not reported so frequently; hopefully, it is the boon of the Indian cuisine that attained much higher temperature than the thermal death point of this pathogen. Since 1996, occurrence of *V. parahaemolyticus* in endemic and epidemic situations has been increasingly reported in many Asian countries including India [28]. Regular rise of ambient and aquatic environment temperature as well as acquiring virulent gene(s) may be associated in abetting such increasing incidences of pathogenic *V. parahaemolyticus* [29]. Warmer sea temperatures (the El Nino effect) have resulted in the emergence of more virulent *V. parahaemolyticus* in USA [30]. Increasing environmental temperature in these areas may facilitate to cope up and propagate the pathogenic strains of this organism.

Epidemiological study revealed that most of the reported outbreaks of *V. parahaemolyticus* infection were due to consumption of raw or insufficiently cooked sea foods especially crustacean and molluscs [4]. The linkage in the transmission of this pathogen through food chain among the consumers of shellfishes in eastern India and probability for incidence of gastroenteritis are seems to be almost similar with the earlier studies because lower income group people of these areas prefer these shellfishes in their daily with dishes that serves the low-cost common dietary affordable protein sources. In modern era of fast food practice, some group of Indian people skip the recommended cooking temperature and time protocol that may accidentally allow entry of this pathogen in food chain. In Indian circumstances, contamination of freshwater fishes at the market level through shellfishes implicated with *V. parahaemolyticus* and subsequently, contamination of other foods in the kitchen by contaminated shellfish brought from markets are believed to be the possible sources of entry of this organism in food chain [31,32]. Factors like improper handling and processing of fish and shellfish at fishing harbors as well as in market are major contributors to contamination by *V. parahaemolyticus* [31]. In Indian context, the physical facilities and infrastructure in all types of fish markets are far from satisfactory. Most fish landing centers and fish markets are old, crowded and have an excess number of traders, even in the passages and without proper infrastructure facilities, thereby resulting in poor fish handling. Most retailers were found selling fish by the roadside without considering either quality or hygiene.

In this investigation, the *tdh*^+^ isolates were screened for the presence of pandemic potential GS gene sequence by employing the GS-PCR and PGS-PCR assay. The GS-PCR elucidates the GS sequence (*toxRS* operon) of *V. parahaemolyticus* that encodes the transmembrane protein involved in the regulation of virulence-associated genes. On comparison sequence in this *toxRS* coding region (1364 bp) between two sets of *V. parahaemolyticus* isolates of O3:K6 serotype (pandemic strain) that was isolated before and after 1995 in different parts of the world, difference was recorded in 7 bases. Moreover, these 7 bases were found conserved in the isolates of such *V. parahaemolyticus* O3:K6 serotype that was isolated after 1995 and termed as *toxRS*/new sequence [20]. With this concept, the sense and antisense primer were designed to amplify the *toxRS*/new sequence by GS-PCR. Similarly, the PGS-PCR yields an amplicon of 235-bp GS sequence of an arbitrarily primed-PCR fragment for 930 bp carried by the pandemic strains of this organism. Accordingly, all the pathogenic (*tdh^+^*) isolates (n=20) were subjected for both PGS-PCR and GS-PCR to determine their pandemic potentials and 11 (6.5%) were found positive in PGS-PCR assay that belonged to crab 6 (7.3%) and shrimp 5 (6%) (Table-1). However, two isolates were found positive in GS-PCR assay and belonged to crab. These two GS-PCR^+^ isolates from crab were also positive in PGS-PCR. The findings indicate that a considerable percentage of shellfishes in these areas were carrying pathogenic *V. parahaemolyticus* that was having well-recognized pandemic potentials in their genotype. The findings also highlight the alarm of public health risk on consumption of improperly processed and cooked shellfishes from such sources. The result also revealed that the PGS-PCR could identify the pandemic potential GS sequence in 6.5% of *tdh*^+^ *V. parahaemolyticus* isolates whereas GS-PCR could do in 1.2% of such isolates. The findings support the preference of PGS-PCR on GS-PCR in detecting the pandemic strains of *V. parahaemolyticus* of saline water origin. The present findings were in accord to the observation of earlier study [21] where a group of pandemic and non-pandemic *V. parahaemolyticus* isolates were extensively examined for identifying the GS pandemic potential gene sequence and concluded that the PGS-PCR assay can be a useful molecular tool not only for identification of pandemic *V. parahaemolyticus* strains but also for direct detection of this organism contaminating food and environmental samples.

Altogether, a constant surveillance on the occurrence of this pathogen in index clinical cases and thereafter, identifying the suspected foods may be beneficial to a large extent to combat the public health problems caused by this pathogen. Further, inculcation of the utmost effective measures, i.e., to educate the people routinely about fundamentals of public health and hygiene will certainly be contributory to reduce the occurrence of health problem with this pathogen and to project a healthy community life.

## Conclusion

This study was envisaged to proximate the occurrence of pathogenic and pandemic *V. parahaemolyticus* in saline water origin shellfishes retailed in coastal parts of Eastern India mainly in and around Kolkata and Bhubaneswar by characterizing their virulence and pandemic genotypes. From the present study, it was concluded that a considerable percentage of shellfishes in these areas are inflicted with pathogenic (*tdh*^+^) (11.9%) and pandemic (6.5%) *V. parahaemolyticus*. This poses public health risk in consumption of improperly processed shellfishes. The health risk arising with cross contamination by such shellfishes to other marine and fresh water market fishes in these areas may be an additional point of risk.

## Authors’ Contributions

This work is the part of M. V. Sc. dissertation work of SP. SP and SCD designed the experiment. SP conducted the experimental work. SCD and AK were involved in scientific discussion and analysis of the data. SCD and AK drafted and revised the manuscript. All authors read and approved the final manuscript.
